# Free-living ciliates as potential reservoirs for eukaryotic parasites: occurrence of a trypanosomatid in the macronucleus of *Euplotes encysticus*

**DOI:** 10.1186/1756-3305-7-203

**Published:** 2014-04-28

**Authors:** Sergei I Fokin, Martina Schrallhammer, Carolina Chiellini, Franco Verni, Giulio Petroni

**Affiliations:** 1 Department of Biology, Protistology-Zoology Unit, University of Pisa, Via A. Volta 4/6, Pisa 56126, Italy; 2 Department of Invertebrate Zoology, St. Petersburg State University, Universitetskaya emb. 7/9, St. Petersburg 198034, Russia; 3 Microbiology, Institute of Biology II, University of Freiburg, Schänzlestraße 1, Freiburg 79104, Germany; 4 Institute of Hydrobiology, Technische Universität Dresden, Zellescher Weg 40, Dresden 01217, Germany; 5 Consiglio per la Ricerca e la Sperimentazione in Agricoltura, Centro di Ricerca per l’Agrobiologia e Pedologia, Piazza D’Azeglio 30, Florence 50121, Italy

**Keywords:** Ciliophora, *Euplotes encysticus*, Endosymbiont, *Herpetomonas*, Host range, *Leptomonas*, Macronucleus, Parasite, *Phytomonas*, Protozoa

## Abstract

**Background:**

Flagellates of the family Trypanosomatidae are obligate endoparasites, which can be found in various hosts. Several genera infect insects and occur as monoxenous parasites especially in representatives of Diptera and Hemiptera. These trypanosomatid flagellates probably share the worldwide distribution of their hosts, which are often infested by large numbers of endoparasites. Traditionally, their taxonomy was based on morphology, host origin, and life cycle. Here we report the characterization of a trypanosomatid infection detected in a protozoan, a ciliate collected from a polluted freshwater pond in a suburb of New Delhi (India).

**Methods:**

Live observations and morphological studies applying light, fluorescence and transmission electron microscopy were conducted. Molecular analyses of host and parasite were performed and used for phylogenetic reconstructions and species (host) or genus level (parasite) identification.

**Results:**

Although the morphological characteristics were not revealing, a high similarity of the trypanosomatids 18S rRNA gene sequence to *Herpetomonas ztiplika* and *Herpetomonas trimorpha* (Kinetoplastida, Trypanosomatidae), both parasites of biting midges (*Culicoides kibunensis* and *Culicoides truncorum,* respectively) allowed the assignment to this genus. The majority of the host population displayed a heavy infection that significantly affected the shape of the host macronucleus, which was the main site of parasite localization. In addition, the growth rate of host cultures, identified as *Euplotes encysticus* according to cell morphology and 18S rRNA gene sequence, was severely impacted by the infection.

**Conclusions:**

The host-parasite system described here represents a recent example of free-living protists acting as environmental reservoirs for parasitic eukaryotic microorganisms.

## Background

Kinetoplastid flagellates belonging to the family Trypanosomatidae are obligate endoparasites of different hosts including vertebrates (*Trypanosoma, Leishmania, Endotrypanum*), plants (*Phytomonas*) and invertebrates [1]. Several genera are frequently found as monoxenous parasites of insects and are especially abundant in members of the orders Diptera and Hemiptera [2-5]. They can be found in high prevalence and apparently share the worldwide distribution of their hosts [4]. Traditionally, their taxonomy was based on morphology, host origin, and life cycle: criteria that were often found to be unreliable. Some trypanosomatid flagellates were isolated from different host species and other hosts were found harboring more than a single species [3,6,7]. These data may suggest a not very explicit host-specificity together with a high physiological plasticity, which could facilitate the establishment of new host-parasite systems [2,8]. Furthermore, it was shown that some of these parasites can exhibit extremely diverse morphologies which may even differ depending on the growth environment, i.e. host or culture medium [1,8,9]. Molecular phylogenetics, based on the 18S rRNA, glycosomal glyceraldehyde phosphate dehydrogenase (gGAPDH) and spliced leader (SL) RNA genes are nowadays considered the method of choice to assess taxonomy and phylogenetic relationships of kinetoplastid flagellates [3-5,10-12].

*Herpetomonas* was one of the first taxonomic divisions within the trypanosomatid flagellates (after *Angomonas* and *Strigomonas*[12]) which was redescribed following a combined approach including molecular and microscopical analyses [5]*.* Also new genera of insect trypanosomatids, i.e. *Sergeia* and *Blechomonas*, have been established based on 18S rRNA and gGAPDH phylogenetic analyses complemented by description of morphological features [10,13].

The genus *Herpetomonas* was previously considered to be polyphyletic. After careful revision, it now represents a monophyletic assemblage comprising 13 species, some of which exhibit extreme morphological polymorphisms. In general, *Herpetomonas* are found as parasites of dipterans, but they have been reported also from predator hemipterans [14], plants [15,16], rats [6], and immunocompromised humans [17].

Transmission pathways of monoxenous insect trypanosomatids to other hosts are still unclear. Hemipterans could acquire the parasites by predation and cannibalism. Possible additional transmission routes are coprophagy and necrophagy [4]. Isolates from plants probably derive either from infection or contamination by insect feces.

There are only a few reports of free-living protists hosting eukaryotic microorganisms evolutionary closely related to pathogens [18-24]. So far, the involved endoparasites have not been molecularly characterized except for the microsporidian infecting *Euplotes woodruffi*[19]. Thus, according to our best knowledge, this characterization of a trypanosomatid flagellate hosted by a ciliated protist belonging to the genus *Euplotes* (Ciliophora, Hypotrichia) is the first molecular description of a member of Trypanosomatidae infecting another protist. Both host and parasite were studied on a morphological and molecular level.

## Methods

### Ciliate isolation and cultivation

*Euplotes* population Ind3 was collected from a heavily eutrophicated freshwater pond near the Yamuna River in the south-east suburb of New Delhi, India (N 28°56’/E 77°29’), in February 2007. Most of the cells harbored an unknown trypanosomatid flagellate localized in the macronucleus (Ma). About 100 cells of infected *Euplotes* sp. were collected and grown as a “laboratory population” in lettuce medium [25] inoculated with *Enterobacter aerogenes.* The establishment of monoclonal cultures from this population failed. Thus, all subsequent investigations were carried out with cells from the laboratory population.

### Light microscopy

Living observations of both host cells and trypanosomatid symbionts were carried out using an Orthoplan Leitz microscope (Hexagon Metrology GmbH Leitz Division, Wetzlar, Germany), equipped with differential interference contrast (DIC), and a Leica DMR microscope (Leica Microsystems GmbH, Wetzlar, Germany). The latter was also used for fluorescence microscopy. Pictures were taken with a Leica DC200 camera.

### Fixation, staining, and electron microscopy

Feulgen staining after fixation in Bouin’s solution [26] was used to reveal the nuclear apparatus of infected *Euplotes* sp. and its trypanosomatid parasites. The flagellate infection in living ciliate cells was observed using ethidium bromide (0.005%) staining followed by fluorescence microscopy. Silver nitrate impregnation for host identification was performed after fixation with Champy’s solution [27]. For electron microscopy, infected cells were processed as described elsewhere [28].

### DNA extraction, amplification of 18S rRNA genes and sequencing

Total DNA extraction was carried out from approximately 50 host cells with NucleoSpinTM Plant DNA Extraction Kit (Macherey-Nagel GmbH & Co., Düren, Germany), following the protocol for mycelium. The almost full-length host and parasite 18S rRNA genes were amplified using specific primer combinations (Additional file 1: Table S1) and Ex Taq PCR reagents (Takara Bio Inc., Otsu, Japan) according to the manufacturer’s instructions.

### Sequence availability and phylogenetic analysis

Characterized 18S rRNA gene sequences are available from the EMBL European Nucleotide Archive (the host population Ind3 [EMBL: HG425175] and its trypanosomatid parasite Ind3 [EMBL: HG425174]).

Phylogenetic analyses were carried out on a subset of 26 *Herpetomonas* (according to [5]) and *Phytomonas* sequences, aligned with the sequence aligner included in the ARB program package [29]. A filter was generated on the used selection to trim them to the same length and retain only positions conserved in at least 50% of the selected sequences. The resulting alignment comprised 1,904 columns.

Tree reconstructions were performed applying Maximum likelihood (PHYML program [30] included in the ARB package) and Bayesian Inference (MrBayes [31]) analysis. For the latter, three different Markov Chain Monte Carlo runs with one cold and three heated chains were performed, running for 1,000,000 generations. The stability of the Maximum likelihood tree was warranted by bootstrap analysis (1,000 pseudoreplicates). A similarity matrix was calculated in ARB.

In addition, the host sequence was aligned in ARB and a similarity matrix of the closest related *Euplotes* sequences was computed.

## Results

### Host identification and effects of parasite infection

**Figure 1:**
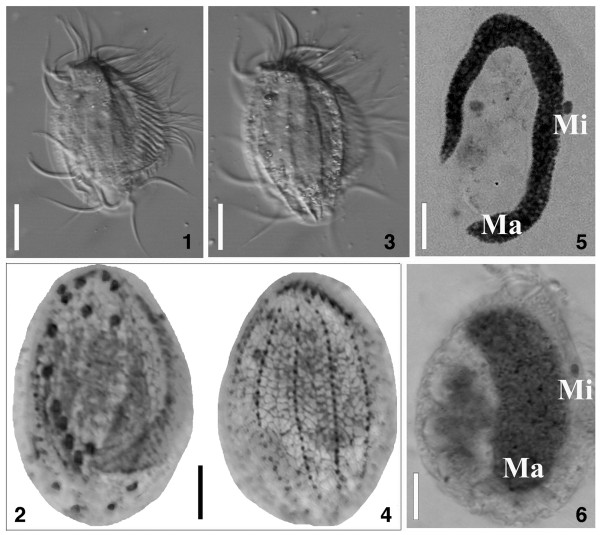
**1–6. General morphology and nuclear apparatus of infected and uninfected *Euplotes encysticus *Ind3. 1**. Ventral view of the ciliate, fixed unstained specimen, DIC contrast. **2**. Ventral view of the ciliate, silver-nitrate impregnation. **3**. Dorsal view of the ciliate, fixed unstained specimen, DIC contrast. **4**. Dorsal view of the ciliate, silver-nitrate impregnation. **5**. Feulgen-staining of uninfected macronucleus. **6**. Feulgen-staining of infected macronucleus. Dramatic changes in organelle shape are well visible. Ma, macronucleus; Mi, micronucleus. Bars = 15 μm **(1, 3)**, 10 μm **(2, 4, 5, 6)**.

The host population was identified as *E. encysticus*, a ciliate morphospecies occurring in freshwater bodies and so far described only from Japan and China [32-34] by classical taxonomic methods of nuclear and cortex preparations (Figure 1. 1–6). According to our best knowledge, this work represents the first report of *E. encysticus* from the Indian subcontinent [35-37]. Phylogenetic analysis supported this morphological identification. The 18S rRNA gene of the Ind3 population showed 100% sequence identity with that of *E. encysticus* EF535728 [34], and 99.94% with *E. encysticus* FJ346569 [38].

The presence of the parasite apparently impaired the growth rate of the host. Attempts to establish monoclonal lines of the infected ciliate failed. Isolated host cells died after a maximum of two divisions. The established laboratory population of *E. encysticus* became extinct after two weeks of investigation. *Euplotes aediculatus* and members of the *Paramecium aurelia* species complex, additionally present in the original water sample, were not infected with the trypanosomatid parasite according to the controlled subset of specimens (for each group: 10 cells examined as living specimen and additional 10–15 cells observed after Feulgen staining).

**Figure 2:**
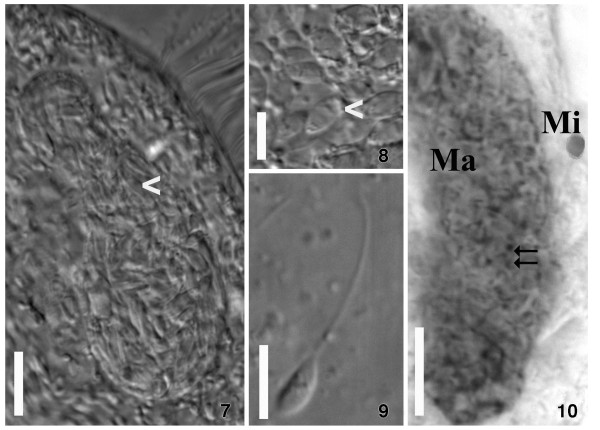
**7–10. Macronucleus of *Euplotes encysticus *infected with trypanosomatid flagellate *Herpetomonas *sp. Ind3. 7**. General view of a heavily infected nucleus. **8**. Part of the nucleus with several trypanosomatids (arrowheads). **9**. Promastigote trypanosomatid released from the crushed macronucleus. **7**–**9**. Living cells, DIC contrast. **10**. Feulgen-staining. Highlighted are the host’s micronucleus (Mi) and macronucleus (Ma), the latter is infected with numerous flagellates; dark spots (arrows) represent nuclei and kinetoplasts of the parasites. Bars = 10 μm **(7, 10)**, 5 μm **(8, 9)**.

*E. encysticus* revealed an infection prevalence of 90% in the studied laboratory population. All infected cells featured macronuclei of unusual shape and size. Instead of the normally occurring relatively slim and C-shaped organelle, the macronucleus of parasitized cells formed a large oval body occupying the main part of the protozoan cytoplasm (Figures 1. 6, 2. 7-10, 3. 11). Each infected macronucleus harbored roughly more than one hundred flagellates (Figures 2. 7-10, 3. 12). In consequence of the infection level, only a small amount of macronulear chromatin could be observed in the Feulgen stained ciliates (Figures 1. 6, 2. 10).

**Figure 3:**
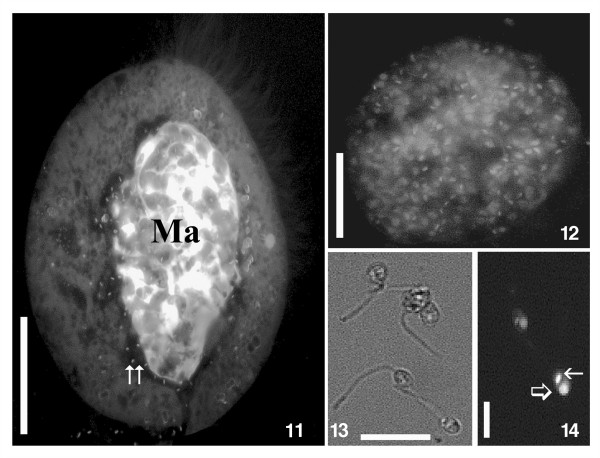
**11–14. Living infected specimens of *Euplotes encysticus *Ind3 and its parasite *Herpetomonas *sp. Ind3 stained with ethidium bromide. 11**. Host cell with a number of parasites released from the infected macronucleus into the cytoplasm (double arrow). **12**. Isolated macronucleus harbouring numerous parasites. **13**. Promastigote stages of the parasite in the medium. **14**. Kinetoplast (small arrow) and nucleus (large arrow) of the parasite. **11**, **12**, **14**. Fluorescent microscopy. **13**. DIC contrast. Bars = 20 μm **(11)**, 15 μm **(12)**, 10 μm **(13)**, 3 μm **(14)**.

**Figure 4:**
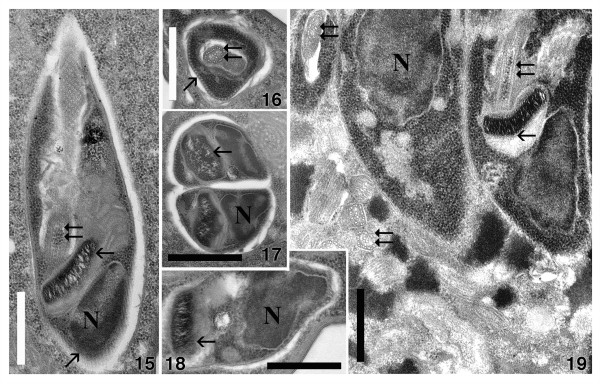
**15–19. Ultrastructure of trypanosomatid flagellate *Herpetomonas *sp. Ind3 infecting *Euplotes encysticus. *15**–**18**. Flagellates harbored in the host cytoplasm. **15**, **18**. Longitudinal sections of flagellates. **16**. Cross-sectioned flagellate through the flagellar pocket; the flagellum, which already bears a paraflagellar rod, is visible. Regularly distributed subpellicular microtubules are also well visible (diagonal arrow). **17**. Cross section of two flagellate cells after fission. **19**. Parasites in the macronucleus of the host. N, nucleus; horizontal arrow, kinetoplast; double arrows, flagellum. Bars = 1 μm.

Inside the host organelle, the flagellates were immotile. After rupture of the macronucleus several parasites started to move vigorously in the culture medium. In a few host cells, low numbers (up to 10–15) of parasites were recorded in cytoplasmatic vacuoles (Figure 4. 15-18), some of those were observed after recently completed cell division (Figure 4. 17).

### Morphological and molecular characterization of the trypanosomatid flagellate

Promastigote forms of the trypanosomatid inside the host cell were characterized by a slightly elongated body (Table 1 and Figures 2. 9, 3. 13, 4. 15). The flagellum originated near the kinetoplast and emerged from the flagellar pocket at the anterior end (Figures 2. 9, 3. 13, 4. 15-19). It was supported by a paraflagellar rod which could be seen in a cross-sectioned flagellum even before it exits the flagellar pocket (Figure 4. 16). The oval nucleus, which was over proportionally large, was situated at the posterior part of the cell, always close to the kinetoplast (Table 1, Figure 4. 14, 15, 17-19). The latter, classically disc-shaped, was located next to the bottom of a deep flagellar pocket. It may occupy a significant part of the cell body (Figure 4. 15, 18, 19) and reached a thickness of 0.30 ± 0.05 μm (Table 1, Figure 4. 15, 18, 19). The flagellate’s plasmalemma was underlaid by subpellicular microtubules with regular spacing (Figure 4. 15, 16). No bacterial endosymbionts were found in the cytoplasm of investigated trypanosomatids.

**Table 1:** **Morphological features and localization within the host organism for some *Herpetomonas *** **and *Leptomonas *** **species**

**Feature**	***Herpetomonas *** **sp. Ind3**	***H. ztiplika***^**a**^	***H. trimorpha***^**a**^	***Leptomonas *** **sp.**	***L. ciliatorum***
Host species	*Euplotes encysticus*	*Culicoides kibunensis, Culicoides truncorum*	*Culicoides truncorum*	*Euplotes* sp.	*Paraholosticha sterkii*
Location	Mainly macronucleus	Hindgut and Malpighian tubules	Malpighian tubules	Only macronucleus	Only macronucleus
Cell length	4.0 ± 0.2	8.9 ± 0.4	8.2 ± 0.3	5	6-8
Cell width	1.3 ± 0.7	2.1 ± 0.1	2.7 ± 1.2	1.7	2-3
Flagellum length	10.2 ± 0.8	10.7 ± 1.1	10.2 ± 2.3	12	10-14
Kinetoplast width	0.30 ± 0.05	0.16 ± 0.07^b^	0.125 ± 0.025^b^	0.18 + 0.0^c^	0.4^c^
Kinetoplast position	Closer to posterior end	Closer to anterior end^b^	Anterior end (mic p, l p)^b^; central (s p)^b^;	Not reported	Nearly central
Nucleus position	Closer to posterior end	Nearly central^b^	Anterior end (mic p)^b^; central (s p, l p)^c^;	Not reported	Close to posterior
Kinetoplast/nucleus position	Close each other	With some distance^b^	Close (mic p, s p)^b^; distant (l p)^b^;	Close each other	Close each other
Posterior end shape	Rounded	Fine pointed^b^	Pointed (mic p)^b^	Rounded	Slightly conical
References	Present study	[8,13]	[9]	[21]	[22]

All measurements given in μm; ^a^data referred to *Herpetomonas* spp. harboured by insect hosts; ^b^data only available from cells in culture medium; ^c^data inferred from published figures; microflagellate (mic), small (s) and long (l) promastigote (p).

**Figure 5:**
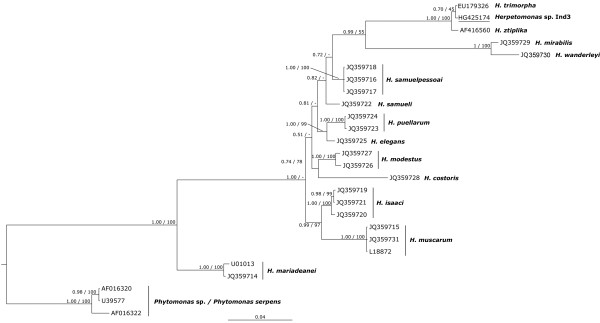
**20. Bayesian tree of members of *Herpetomonas *and *Phytomonas.*** The tree was built on the unmodified character matrix of 23 *Herpetomonas* and 3 *Phytomonas* sequences (1,904 columns) employing the GTR + I + G model. Numbers associated to each node correspond to posterior probability and Maximum Likelihood bootstrap values. The scale bar represents an estimated sequence divergence of 4%. Underlined is the sequence of the trypanosomatid flagellate of *Euplotes encysticus* Ind3.

The phylogenetic reconstructions presented here are in accordance with the recent redescription of the genus *Herpetomonas*[5]. All 13 *Herpetomonas* species were recovered with some minor differences in the branching order of the computed tree (Figure 5. 20) in respect to published results [5].

A similarity value (Additional file 2: Table S2) of 99.42% and 99.84%, respectively, is shared among the trypanosomatid 18S rRNA gene sequence described here and *Herpetomonas ztiplika* AY308760 [8] and *Herpetomonas trimorpha* EU179326 [9]. All three sequences formed a highly stable association (Figure 5. 20), but the affiliations within the group are less well supported.

## Discussion

The role of free-living protists as reservoir hosts or even vectors for pathogens has been underestimated for a long time. Their significance in the genesis of several bacterial pathologies has been re-evaluated after disease outbreaks caused by protist-born microorganisms, i.e. *Legionella pneumophila*, the etiological agent of the Legionnaires’ disease, and other amoebae-hosted pathogens [39,40]. In the last decade, many associations between free-living protists and bacteria phylogenetically closely related to human or animal pathogens have been described. The most compelling examples refer to the families *Francisellaceae* and *Rickettsiaceae.* A novel *Francisella* subspecies harbored by a ciliate [41] and many “basal rickettsiae” from amoebae [42], several ciliates [43-46] and green algae [47] have been described.

Despite the common capabilities of many free-living protists to host other eukaryotic microorganisms like many different kinds of eukaryotic “algae” [48-50], there are only a few reports of free-living ciliates hosting eukaryotic microorganisms belonging to evolutionary lineages that comprehend also human and/or animal pathogens. These include the microsporidians *Ciliatosporidium platyophryae* in *Platyophrya terricola*[18], and *Euplotespora binucleata* in *Euplotes woodruffi*[19], as well as different trypanosomatid species described in the macronuclei of *Paramecium trichium* (the younger synonym of *Paramecium putrinum*) [20], *Euplotes* sp. [21] and *Paraholosticha sterkii*[22].

None of these organisms have been characterized at the molecular level with the exception of *Euplotespora binucleata*[19]. These neglected reports [18-22] and the characterization presented here suggest that at least some free-living protists may act as a reservoir for parasitic ones. The possible capability of the latter to also parasitize higher organisms is a matter that, in our opinion, should deserve further attention. In this context, the extremely limited 18S rRNA distance (less than 0.58%) among *Herpetomonas* strains retrieved from midges, i.e. *H. ztiplika* and *H. trimorpha,* and the one characterized in the present report is particularly intriguing. Various transmission routes (e.g. predation, cannibalism, coprophagy and fecal contamination) for monoxenous insect trypanosomatids have been reported [4]. The general mechanism is based on uptake of infected organisms or material. It remains unclear how ciliates can acquire an infection but a plausible scenario involves the aquatic larval stage of infected insect hosts. The host flies of *H. ztiplika* and *H. trimorpha* (family Ceratopogonidae) comprise a semi-aquatic life style with their larval stages developing in bodies of freshwater. In these habitats also free living protozoans including ciliates do occur. Thus, an encounter of e.g. *Euplotes* with infected larvae is possible. Like many other ciliates, *Euplotes* is a filter feeder and might haphazardly ingest infected organic material or released trypanosomatid flagellates.

It has already been shown that the existence of extensive size and shape polymorphism within a single trypanosomatid species according to cultivation conditions strongly impairs the resolution power of those characters for identification and taxonomy [1,8,9,15]. The consequential conflict between traditional taxonomy and molecular phylogeny is well known also for the genus *Herpetomonas* and resulted in the recent redescription of this taxon, based mainly on molecular data [5]. For the affiliation of the newly found trypanosomatid from *E. encysticus,* application of morphological features was not feasible. According to morphology, the novel trypanosomatid shares similarities with some leptomonads described from other ciliates (size and shape of promastigote, kinetoplast width and position; Table 1) rather than with members of *Herpetomonas.* In particular *H. ztiplika* or *H. trimorpha*, which are its nearest relatives according to molecular analyses (Figure 5. 20), share few morphological characteristics with the trypanosomatid described here (Table 1).

In contrast to morphology, molecular data demonstrate a strong affiliation of the newly characterized trypanosomatid to the genus *Herpetomonas* and, in particular, to *H. trimorpha* and *H. ztiplika* species; indeed the three organisms show an 18S rRNA gene sequence identity higher than 99.4% among themselves. Nevertheless, as far as no additional phylogenetic marker e.g. the gGAPDH gene sequence was obtained, an unambiguous affiliation to one of the mentioned *Herpetomonas* species or the establishment of a new species is not feasible.

Interestingly, the isolates of *H. ztiplika*[8] and *H. trimorpha* have the same geographic origin. Both have been recovered from biting midges collected in the Milovický forest in the vicinity of Mikulov, Czech Republic, whereas the host of the endoparasite described in this study was collected in a suburb of New Delhi, India. This observation adds to other reports of cosmopolitan trypanosomatid species [1,4,5,7] and the limited value of geographic and host origin for trypanosomatid and especially *Herpetomonas* taxonomy.

Morphological similarities between the newly described *Herpetomonas* sp. Ind3 and previously described ciliate-born leptomonads can be explained either as adaptation to similar host organisms (ciliated protist) and cell compartments (macronucleus) or to the misclassification of previously described ciliate-born trypanosomatids; only the molecular characterization of other trypanosomatids from ciliates will help to clarify this point.

Considering the present and previously reported cases, it is quite possible that some of the ciliates (eventually with the exception of *Paraholosticha sterkii*) are not the main host for the flagellates. This view is supported by the close phylogenetic relationship of the here described *Herpetomonas* sp. Ind3 to monoxenous parasites of dipterans and the severely impaired growth rate of infected *E. encysticus* Ind3.

## Conclusions

Based on the results presented here we demonstrate that free-living protists can act at least as temporary environmental reservoirs for parasitic eukaryotic microorganisms. The capacity (or susceptibility) of ciliates for infections with “arthropod-specific” parasites has recently been reported for members of the obligate intracellular *Rickettsiaceae* (*Alphaproteobacteria*) [51-53]. Especially noteworthy is the bacterium ‘*Candidatus* Megaira polyxenophila’ [43] which has been frequently found in a broad range of diverse protist hosts.

Extensive screening efforts revealed that at least some trypanosomatid species may infect a wide range of hosts, in some cases even spanning different insect orders [6], which lead to the abolishment of the “one host – one parasite” paradigm. So far, trypanosomatid flagellates have been regarded as typical dipteran and hemipteran parasites. This pattern might change when more infected ciliates are described in the future.

It has been stated that trypanosomatid flagellates exhibit a low host specificity which might be caused by a high physiological plasticity and allow the establishment of new host-parasite systems [2,8]. In the case of *Herpetomonas,* this plasticity apparently allows a host range encompassing not only of different insect orders but also unicellular host organisms. Furthermore, it permits an endoparasitic as well as an intracellular, intramacronuclear lifestyle.

## Competing interests

The authors declare that they have no competing interests.

## Author’s contributions

SIF and GP designed the study; sampling and isolation were carried out by SIF as well as the microscopical analysis, VF contributed to the microscopical analysis; primer design was done by GP; MS performed the phylogenetic reconstructions; SIF, MS and CC conducted the literature review and drafted the manuscript. All authors read and approved the final manuscript.

## Supplementary Material

Additional file 1: Table S1 Oligonucleotides used in the present work. Click here for file

Additional file 2: Table S2 18S rRNA similarity matrix of *Herpetomonas* species. Click here for file
