# Supplementary material for: Free-living ciliates as potential reservoirs for eukaryotic parasites: occurrence of a trypanosomatid in the macronucleus of Euplotes encysticus
Source: Parasit Vectors. 2014 Apr 28;7:203. doi: 10.1186/1756-3305-7-203 (PMC4022238; doi:10.1186/1756-3305-7-203)
Supplement: Additional file 1: Table S1 — Oligonucleotides used in the present work. [file 1756-3305-7-203-S1.docx]

**Table S1. Oligonucleotides used in the present work.**

| **Primer Name** | **Direction** | **Position^a^** | ***5′ – 3′* Sequence** | **Specificity** | **Application** | **Reference** |
| --- | --- | --- | --- | --- | --- | --- |
| F6 Euk | Forward | 6 | AAYCTGGTTGATYYTGCCAG | Eukaryota | Amplification (host) | [1] |
| F9 Kineto | Forward | 9 | TCTGGTTGATTCTGCCAG | Kinetoplastida | Amplification (parasite) | present work |
| R536 | Reverse | 536 | CTGGAATTACCGCGGCTG | Eukaryota | Sequencing (host) | [2] |
| R536 Kineto | Reverse | 536 | CTGGAATTACCGCGGGTG | Kinetoplastida | Sequencing (parasite) | present work |
| F783 Kineto | Forward | 783 | GATGATTAGAGACCATTGTA | Kinetoplastida | Sequencing (parasite) | present work |
| F919 | Forward | 919 | ATTGACGGAAGGGCACCA | Eukaryota | Sequencing (host) | [2] |
| F919 Kineto | Forward | 919 | ATTGACGGAATGGCACCA | Kinetoplastida | Sequencing (parasite) | present work |
| R1052 | Reverse | 1052 | AACTAAGAACGGCCATGCA | Eukaryota | Sequencing (host) | [2] |
| R1052 Kineto | Reverse | 1052 | GACCAAAAGCGGCCATGC | Kinetoplastida | Sequencing (parasite) | present work |
| F1097 Kineto | Forward | 1097 | GTCAACGGACGA GAT CC | Kinetoplastida | Sequencing (parasite) | present work |
| R1505 Euglen | Reverse | 1505 | AGGTTCACCTACAGCAAC | Euglenozoa | Amplification (parasite) | present work |
| R1513 Hypo | Reverse | 1513 | TGATCCATCTGCAGGTTC | Hypotrichia | Amplification (host) | [3] |

^a^ Referred to the corresponding position in *E. coli* SSU rRNA.

Polymerase chain reactions were carried out at 94 °C for 3 min followed by 35 cycles of 94 °C for 30 s, 50 °C for 30 s and 72 °C for 2 min 30 s, with a final extension step at 72 °C for 10 min.

Additional references:

1. Modeo L, Petroni G, Rosati G, Montagnes D: **A multidisciplinary approach to describe protists: redescriptions of *Novistrombidium testaceum* Anigstein 1914 and *Strombidium inclinatum* Montagnes, Taylor, and Lynn 1990 (Ciliophora, Oligotrichia).** *J Eukaryot Microbiol* 2003, **50:**175–189.

2. Rosati G, Modeo L, Melai M, Petroni G, Verni F: **A multidisciplinary approach to describe protists: a morphological, ultrastructural, and molecular study on *Peritromus kahli* Villeneuve-Brachon, 1940 (Ciliophora, Heterotrichea).** *J Eukaryot Microbiol* 2004, **51:**49–59.

3. Petroni G, Dini F, Verni F, Rosati G: **A molecular approach to the tangled intrageneric relationships underlying phylogeny in *Euplotes* (Ciliophora, Spirotrichea).** *Mol Phylogenet Evol* 2002, **22:**118–30.
