# Supplementary material for: Free-living ciliates as potential reservoirs for eukaryotic parasites: occurrence of a trypanosomatid in the macronucleus of Euplotes encysticus
Source: Parasit Vectors. 2014 Apr 28;7:203. doi: 10.1186/1756-3305-7-203 (PMC4022238; doi:10.1186/1756-3305-7-203)
Supplement: Additional file 2: Table S2 — 18S rRNA similarity matrix of Herpetomonas species. [file 1756-3305-7-203-S2.docx]

**Table S2. 18S rRNA similarity matrix of *Herpetomonas* species.**

| **Organism** | **1** | **2** | **3** | **4** | **5** | **6** | **7** | **8** | **9** | **10** | **11** | **12** | **13** | **14** | **15** | **16** | **17** | **18** | **19** | **20** | **21** | **22** | **23** |
| --- | --- | --- | --- | --- | --- | --- | --- | --- | --- | --- | --- | --- | --- | --- | --- | --- | --- | --- | --- | --- | --- | --- | --- |
| *H. pessoai* JQ359718 | 100 |  |  |  |  |  |  |  |  |  |  |  |  |  |  |  |  |  |  |  |  |  |  |
| *H. pessoai* JQ359716 | 100 | 100 |  |  |  |  |  |  |  |  |  |  |  |  |  |  |  |  |  |  |  |  |  |
| *H. pessoai* JQ359717 | 100 | 100 | 100 |  |  |  |  |  |  |  |  |  |  |  |  |  |  |  |  |  |  |  |  |
| *H. samueli* JQ359722 | 98.47 | 98.47 | 98.47 | 100 |  |  |  |  |  |  |  |  |  |  |  |  |  |  |  |  |  |  |  |
| *H. puellarum* JQ359723 | 97.41 | 97.41 | 97.41 | 97.57 | 100 |  |  |  |  |  |  |  |  |  |  |  |  |  |  |  |  |  |  |
| *H. puellarum*  JQ359724 | 97.47 | 97.47 | 97.47 | 97.62 | 99.95 | 100 |  |  |  |  |  |  |  |  |  |  |  |  |  |  |  |  |  |
| *H. elegans* JQ359725 | 98.05 | 98.05 | 98.05 | 98.26 | 98.68 | 98.73 | 100 |  |  |  |  |  |  |  |  |  |  |  |  |  |  |  |  |
| *H. modestus* JQ359726 | 97.47 | 97.47 | 97.47 | 97.47 | 97.09 | 97.15 | 97.73 | 100 |  |  |  |  |  |  |  |  |  |  |  |  |  |  |  |
| *H. modestus*  JQ359727 | 97.53 | 97.53 | 97.53 | 97.52 | 97.25 | 97.31 | 97.79 | 99.53 | 100 |  |  |  |  |  |  |  |  |  |  |  |  |  |  |
| *H. costoris*  JQ359728 | 95.31 | 95.31 | 95.31 | 95.73 | 95.45 | 95.51 | 96.04 | 95.83 | 96.00 | 100 |  |  |  |  |  |  |  |  |  |  |  |  |  |
| *H. isaaci*  JQ359719 | 97.10 | 97.10 | 97.10 | 97.52 | 97.09 | 97.09 | 97.41 | 97.15 | 97.15 | 95.67 | 100 |  |  |  |  |  |  |  |  |  |  |  |  |
| *H. isaaci* JQ359721 | 97.15 | 97.15 | 97.15 | 97.57 | 97.14 | 97.14 | 97.47 | 97.20 | 97.20 | 95.72 | 99.95 | 100 |  |  |  |  |  |  |  |  |  |  |  |
| *H. isaaci* JQ359720 | 97.10 | 97.10 | 97.10 | 97.52 | 97.04 | 97.04 | 97.36 | 97.15 | 97.15 | 95.62 | 99.68 | 99.74 | 100 |  |  |  |  |  |  |  |  |  |  |
| *H. muscarum* JQ359715 | 96.14 | 96.14 | 96.14 | 96.19 | 95.86 | 95.86 | 96.13 | 96.13 | 96.03 | 94.27 | 97.14 | 97.19 | 97.14 | 100 |  |  |  |  |  |  |  |  |  |
| *H. muscarum* JQ359731 | 96.14 | 96.14 | 96.14 | 96.19 | 95.86 | 95.86 | 96.13 | 96.13 | 96.03 | 94.27 | 97.14 | 97.19 | 97.14 | 100 | 100 |  |  |  |  |  |  |  |  |
| *H. muscarum* L18872 | 96.13 | 96.13 | 96.13 | 96.18 | 95.85 | 95.86 | 96.13 | 96.13 | 96.03 | 94.27 | 97.14 | 97.19 | 97.14 | 100 | 100 | 100 |  |  |  |  |  |  |  |
| *H. trimorpha* EU179326 | 94.35 | 94.35 | 94.35 | 94.24 | 94.01 | 94.07 | 93.86 | 93.49 | 93.45 | 92.38 | 94.13 | 94.13 | 93.97 | 93.41 | 93.41 | 93.41 | 100 |  |  |  |  |  |  |
| *H. ztiplika* AF416560 | 94.45 | 94.45 | 94.45 | 94.19 | 93.96 | 94.01 | 93.81 | 93.44 | 93.50 | 92.43 | 93.92 | 93.92 | 93.76 | 93.36 | 93.36 | 93.36 | 99.47 | 100 |  |  |  |  |  |
| ***Herpetomonas* sp. Ind3 HG425174** | 94.40 | 94.40 | 94.40 | 94.29 | 94.06 | 94.12 | 93.92 | 93.55 | 93.50 | 92.43 | 94.18 | 94.18 | 94.02 | 93.47 | 93.47 | 93.46 | 99.84 | 99.42 | 100 |  |  |  |  |
| *H. wanderleyi* JQ359730 | 92.80 | 92.80 | 92.80 | 92.79 | 92.50 | 92.45 | 92.36 | 91.94 | 92.11 | 91.20 | 92.36 | 92.42 | 92.42 | 92.01 | 92.01 | 92.01 | 91.83 | 91.83 | 91.88 | 100 |  |  |  |
| *H. mirabilis* JQ359729 | 93.43 | 93.43 | 93.43 | 93.21 | 92.88 | 92.83 | 92.94 | 92.68 | 92.79 | 91.72 | 92.94 | 92.99 | 92.94 | 92.59 | 92.59 | 92.59 | 92.19 | 92.25 | 92.25 | 98.25 | 100 |  |  |
| *H. mariadeanei* JQ359714 | 92.83 | 92.83 | 92.83 | 92.50 | 92.65 | 92.70 | 92.66 | 92.71 | 92.72 | 91.59 | 93.19 | 93.19 | 93.35 | 92.52 | 92.52 | 92.51 | 91.26 | 91.00 | 91.26 | 90.48 | 90.75 | 100 |  |
| *H. mariadeanei* U01013 | 92.60 | 92.60 | 92.60 | 92.32 | 92.47 | 92.53 | 92.49 | 92.53 | 92.54 | 91.41 | 93.02 | 93.02 | 93.18 | 92.29 | 92.29 | 92.29 | 91.09 | 90.82 | 91.09 | 90.25 | 90.52 | 99.68 | 100 |

Highlighted in bold is the sequence of the trypanosomatid flagellate of *Euplotes encysticus* Ind3.
